# The Roles of Lipid Metabolism in the Pathogenesis of Chronic Diseases in the Elderly

**DOI:** 10.3390/nu15153433

**Published:** 2023-08-03

**Authors:** Rui Song, Mengxiao Hu, Xiyu Qin, Lili Qiu, Pengjie Wang, Xiaoxu Zhang, Rong Liu, Xiaoyu Wang

**Affiliations:** 1 College of Food Science & Nutritional Engineering, China Agricultural University, Beijing 100083, China; songrui_123@cau.edu.cn (R.S.); humengxiao@cau.edu.cn (M.H.); qinxiyu@cau.edu.cn (X.Q.); qiulily@cau.edu.cn (L.Q.); 2 Key Laboratory of Precision Nutrition and Food Quality, Department of Nutrition and Health, China Agricultural University, Beijing 100193, China; wpj1019@cau.edu.cn (P.W.); xiaoxuzhang0220@cau.edu.cn (X.Z.); liurong@cau.edu.cn (R.L.)

**Keywords:** elderly, lipid, digestion, absorption, metabolism, chronic diseases

## Abstract

Lipid metabolism plays crucial roles in cellular processes such as hormone synthesis, energy production, and fat storage. Older adults are at risk of the dysregulation of lipid metabolism, which is associated with progressive declines in the physiological function of various organs. With advancing age, digestion and absorption commonly change, thereby resulting in decreased nutrient uptake. However, in the elderly population, the accumulation of excess fat becomes more pronounced due to a decline in the body’s capacity to utilize lipids effectively. This is characterized by enhanced adipocyte synthesis and reduced breakdown, along with diminished peripheral tissue utilization capacity. Excessive lipid accumulation in the body, which manifests as hyperlipidemia and accumulated visceral fat, is linked to several chronic lipid-related diseases, including cardiovascular disease, type 2 diabetes, obesity, and nonalcoholic fatty liver disease. This review provides a summary of the altered lipid metabolism during aging, including lipid digestion, absorption, anabolism, and catabolism, as well as their associations with age-related chronic diseases, which aids in developing nutritional interventions for older adults to prevent or alleviate age-related chronic diseases.

## 1. Introduction

Globally, the proportion of elderly people aged 65 years or over continues to increase. According to the United Nations statistics, in 2019, the elderly population aged 65 years or over accounted for 9% of the total population, and this proportion is projected to increase to 16% by 2050 and surpass 23% by 2100 [1]. The number of people aged 80 years or over is growing even faster as a consequence of increased global life expectancy. Age is a major risk factor for chronic diseases, and more than 50% of the elderly suffer from at least one kind of chronic disease. For example, cardiovascular disease (CVD) affects up to 70% of the elderly population, and diabetes affects about 20% of the elderly population [2,3,4]. The high cost of these chronic diseases brings major personal and public health burdens, which will even get worse in the future. Therefore, it is advisable to prevent the occurrence of these diseases as early as possible. These diseases are commonly accompanied by dyslipidemia and insulin resistance that result from lipid metabolism disorders, thus indicating that modulating lipid metabolism may be an effective way to prevent or alleviate these chronic diseases.

The dysregulation of lipid metabolism contributes to many age-related chronic diseases [5]. Lipid metabolism is a complex biochemical reaction, which refers to the process of the digestion, absorption, synthesis, and catabolism of lipids. As a result of aging, all gastrointestinal processes (such as movement, enzyme, hormone release, and so on) are altered, which in turn affects digestion and absorption, thereby leading to reduced nutrient uptake. Postprandial lipemia (PPL) is the phenomenon of elevated post-meal blood lipids, which typically decreases over time. Despite the reduced intestinal uptake, the excessive and prolonged PPL observed in the elderly suggests the sustained elevation of lipid concentrations after meals, thus indicating an imbalance in blood lipid circulation. The strict regulation of lipid metabolism in vivo after lipid uptake is crucial for maintaining lipid homeostasis. Changes in lipid synthesis and catabolism that occur with aging lead to abnormal lipid utilization in tissues. Blood lipid and other chronic disease precursors arise as a result of lipid metabolic disorders, which eventually develop into several chronic diseases, such as CVD, type 2 diabetes (T2D), nonalcoholic fatty liver disease (NAFLD), obesity, and so on [6]. Therefore, the disturbed lipid homeostasis in the elderly highlights the significance of the strict regulation of lipid metabolism in maintaining overall health.

This review covers the changes in lipid metabolism during aging, including lipid digestion and absorption, as well as the anabolism and catabolism capacity of triglycerides (TGs) and cholesterol and the pathogenesis of several lipid-related diseases in the elderly. Our aim is to gain insight into the lipid uptake and utilization patterns in the elderly to provide personalized nutritional recommendations for older adults, thereby preventing or alleviating lipid-related chronic diseases to promote healthy aging.

## 2. Disorder of Lipid Digestion and Absorption in the Elderly

**Figure 1:**
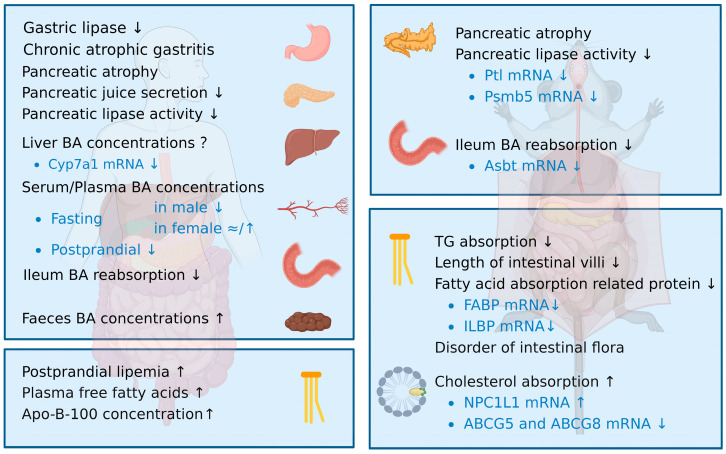
Age-related changes in lipid digestion and absorption. The image on the left depicts changes in the digestive tracts of elderly people, and the image on the right shows changes in the digestive tracts of aging rodents. Alterations in digestive function occur in the stomach, pancreas, liver, blood, intestines, and feces. Both TG and cholesterol absorption that occur in the intestine are described. ↑ means studies showing a significant increase; ↓ means studies showing a significant decrease; ≈ means studies showing no significant decrease; ? means this result has not been reported. Figure 1 was created using Biorender with permission for publication from Biorender.com (accessed on 9 July 2023).

Digestion and absorption play critical roles in whole-body lipid metabolism by providing lipid digestive products as metabolic substrates. Lipid digestion primarily occurs through digestive enzymes secreted by the pancreas, followed by absorption in the small intestine, which is the main pathway for exogenous lipids to enter the body. The excessive intake of dietary fats can lead to lipid accumulation in the body, which promotes the development of chronic lipid-related diseases. Age-related changes in lipid digestion may be attributed to degeneration of the digestive tract function, such as atrophied organs, reduced pancreatic secretion, insufficient lipase production, and decreased bile acid (BA) concentrations [7]. In addition, current studies have indicated that lipid absorption may decrease, while cholesterol absorption increases with age [8] (Figure 1). Therefore, lipid digestion and absorption processing may become a new target for dietary interventions to prevent dyslipidemia in the elderly and to treat lipid-related diseases.

### 2.1. Age-Related Changes in Lipid Digestion

Lipid digestion is an intricate process involving multiple stages. Lipid digestion begins in the mouth and continues in the stomach, with lingual lipase playing a minor role and gastric lipase contributing 10–30% to the overall lipid breakdown in adults [9]. Subsequently, the partially hydrolyzed lipids enter the duodenum as small lipid droplets (LDs). Since dietary lipids are hydrophobic, and lipase is hydrophilic, further emulsification takes place through the action of bile salts to increase the surface area for efficient digestion. These micelles can pass through the unstirred water layer, thereby allowing for lipid absorption to occur [10].

#### 2.1.1. Gastric Lipase Activity Decreases with Age

The contribution of the stomach to lipid digestion is reduced in comparison to younger individuals and is further diminished in elderly individuals with gastric atrophy or those with the chronic use of acid suppression medication. Previous studies have shown a significant negative correlation between age and gastric lipase activity; however, the mechanism behind this phenomenon has not been elucidated [11]. Gastric lipase activity is particularly stable at a low pH [12], but the high prevalence of chronic atrophic gastritis and the widespread use of proton pump inhibitor drugs in the elderly population could result in a decline in gastric acid secretion, which may affect gastric lipase activity [13].

#### 2.1.2. Pancreatic Function Declines with Age Due to Decreased Pancreatic Lipase Expression

The pancreas is an important organ for digestion, and morphological changes occur with age. Magnetic resonance imaging (MRI) was used to scan the morphological characteristics of the pancreas in over two hundred healthy participants, which revealed that the pancreases of elderly subjects showed signs of atrophy. Moreover, the critical age range for pancreatic morphological changes was found to be 40 years old and 60 years old [14]. Consistently, MRI demonstrated increased pancreatic atrophy, lobulation, and steatosis in aged mice [15].

Along with pancreas atrophy, its function also declines. Numerous studies have demonstrated a positive correlation between physical aging and the decline in the exocrine function of the pancreas [14]. Specifically, the lower secretory flow rate of the pancreatic juice in humans indicated a significant reduction in the secretion ability with aging [16]. Moreover, the time point for changes in pancreatic exocrine function was found to be consistent with that of morphological changes, with degeneration occurring at 43 years of age [17].

The primary age-related change in pancreatic function is the decline of the pancreatic lipase activity. In healthy adults, pancreatic lipase is responsible for digesting approximately three-quarters of dietary TGs. However, its digestive efficiency decreases with aging [18]. The concentration and secretion volume of lipase showed a gradual decline in participants after the age of 30 [17]. Researchers collected duodenal fluid to evaluate the output and concentration of lipases after continuous intravenous infusions of irritants in elderly patients without pancreatic diseases. It was found that lipase concentrations declined significantly, by about 15%, in the older group compared to the younger group. Additionally, it was observed that lipase output levels decreased substantially by 45% [19]. Similar results were also observed in aging mice, in which the activity of the pancreatic lipase decreased significantly [20].

Pancreatic lipase activity may be reduced due to the accumulation of unfolded enzymes in the pancreas. The protein expressions of pancreatic lipases in elderly mice are consistent with those in younger mice [21]. As with other enzymes, pancreatic lipase is a linear polypeptide chain that must be folded into a unique 3D structure to perform its activity and physiological role [22]. Interestingly, it was found that the level of proteasome subunit beta type 5 (Psmb5), which facilitates the elimination of enzymes that have not been folded into specific structures, decreased significantly in 25-month-old mice compared to 3-month-old mice. Therefore, the accumulation of nonfunctional enzymes may affect the synthesis or secretion of effective lipases [21].

#### 2.1.3. Aging Is Mainly Associated with a Decline in Bile Acid Levels Due to the Lower Ileum Reabsorption

BA plays a crucial role in facilitating the digestion and absorption of dietary lipids. This is due to the amphiphilic structure of BA, which allows it to function as an emulsifier [23]. Age-related changes in the composition of BA have been studied in both humans and animals. Although the changes varied depending on certain factors such as gender, BA levels showed a consistent decrease with age [10].

In the fasting state, the plasma concentrations of BA decreased with age in men, but they remained constant or showed a slight increase in women. Similarly, in the postprandial state, the responses of BAs were weaker in the elderly compared to young people. The serum BA levels in healthy young subjects increased rapidly after a meal, whereas elderly individuals showed a smaller increase in their serum BA levels after a meal [24]. For this reason, there is some indirect evidence to suggest that intestinal BA reabsorption becomes reduced in older adults. This hypothesis was further supported by the higher fecal concentrations of BAs in older adults, thereby indicating decreased reabsorption in the ileum [25]. Additionally, during the enterohepatic circulation in the elderly, a higher rate of deoxycholic acid (DCA) was found in the colon, which is a metabolic byproduct of cholic acid (CA) transformation [26]. This finding suggests a reduction in the reabsorption of BA in the ileum among older adults, thereby leading to an increased CA load in the colon.

As was consistent with human studies, the reabsorption of BA in the ileum of aging mice was similarly reduced, with about a 50% lower absorption of taurocholate than in younger mice. This was supported by the reduced gene expression of apical sodium-dependent bile acid transporter (Asbt), which is the key transporter regulating BA uptake in the intestinal apical membrane [27]. These observations suggest that the decreased expression of Asbt potentially acts as a rate-determining step in BA reabsorption.

### 2.2. Age-Related Changes in Lipid Absorption

After digestion, lipids are absorbed in the lower part of the duodenum and the upper part of the jejunum. The lipid hydrolysis products form micelles, which contain free fatty acids (FFA), monoacylglycerols, free cholesterol, and so on. These micelles could diffuse through the unstirred water layer and eventually release their lipid contents into the enterocytes. Typically, fatty acid translocase (CD36/FAT), fatty acid transporter 4 (FATP4), and membrane-associated fatty acid binding protein (FABP) transporters mediate TG absorption, while Niemann–Pick C1-like 1 (NPCIL1) mediates cholesterol absorption [28,29].

#### 2.2.1. Triglyceride Absorption Decreases with Age

The absorption of dietary fat initiates an increase in the plasma TG concentration, and the resultant increase in chylomicron and very low-density lipoproteins (VLDL) in the blood in the postprandial state is known as PPL. Increased PPL has been typically observed in older adults [30,31]. Research projects have shown that PPL in older adults increases by about 130% compared to younger adults, which is associated with higher plasma FFA and apolipoprotein B-100 concentrations and a lower rate of oxidation of the ingested lipid [32]. By lowering dietary fat intake, PPL can be reduced by about 50%, and FFA can be reduced by about 190% in older adults [33]. However, it should be noted that, while PPL reflects absorption, its true significance is in the concentration of lipids in the bloodstream. Therefore, an increase in PPL in older adults may also be attributed to a decreased efficiency of lipid utilization.

Due to the significant time and cost required to validate studies in humans, the majority of aging data comes from shorter-lived rodents. The investigations on lipid absorption in aged mice have consistently reported decreased outcomes with humans. Researchers investigated changes in lipid absorption in 3- and 25-month-old mice and found that, after the oral administration of soybean oil, the increase in serum TG levels in older mice was significantly reduced [21]. Nevertheless, only a few studies have obtained similar results, and further research is needed to confirm this finding. There are several known mechanisms that could explain the decline of the absorption capacity in older individuals. Morphologically, changes in the small intestine may lead to a decline in nutrients absorption in aging mice, as the length of intestinal villi decreases with age [34]. Some researchers have also suggested that the decline in intestinal lipid intake may be associated with a reduction in the abundance of fatty acid (FA)-absorption-related proteins during aging. For example, studies have shown that the abundance of intestinal FABP and ileal lipid-binding protein (ILBP) decreases in aging rats [35]. Moreover, aging is often accompanied by intestinal flora disorders, and previous studies have indicated that the small intestinal microbiota could regulate the digestion and absorption of dietary fat in hosts [36,37].

In brief, the efficiency of dietary lipids absorption affects lipid homeostasis. However, the lipid absorption capacity in older adults remains unknown. As previously discussed, several known mechanisms may contribute to the decline in the absorption capacity in the elderly. However, further research is needed to determine the impact of aging on lipid absorption, including the absorption time and efficiency.

#### 2.2.2. Cholesterol Absorption Increases with Age

Numerous investigations have indicated that the absorption of cholesterol will increase with age. Research has indicated that cholesterol absorption increased with age in mice [38]. In addition, a mouse model has shown that the expressions of the sterol efflux transporters adenosine triphosphate-binding cassette transporters G5 and G8 (ABCG5 and ABCG8) in the jejunum and ileum were down-regulated, and the expression of sterol efflux transporter NPC1L1 in duodenum and jejunum was up-regulated during aging [39]. The above changes may be related to the increased intestinal cholesterol absorption in aged mice. At present, the successful prescription drugs on the market treating hypercholesterolemia work by inhibiting NPC1L1 to reduce the cholesterol level in the blood. In addition, dietary phytosterol or stanol supplements could also treat hypercholesterolemia [40]. Further research is needed to investigate the efficacy and safety of these interventions, as well as to explore more effective interventions that may improve cholesterol homeostasis in aging populations.

## 3. Disorder of Lipid Anabolism and Catabolism in the Elderly

Lipid anabolism and catabolism are fundamental processes involved in the biosynthesis and degradation of FAs, TGs, cholesterol, and other lipid substances [41]. The liver is the most important organ for lipid synthesis and catabolism. However, lipids cannot be stored in hepatocytes, and adipose cells act as the main reservoir for fat storage. Lipids must be transported from where they are absorbed or synthesized to where they are used or stored. However, as lipids are insoluble in water, they have to bind to proteins to form lipoproteins before being transported. Thus, plasma lipoproteins are essential for lipid circulation throughout the body. It is important to note that complex changes occur in lipid metabolism during aging, which can contribute to the development of lipid-related diseases [42].

### 3.1. Disorder of Plasma Lipoprotein Metabolism during Aging

The dysregulation of the lipid metabolism in older adults is often reflected by changes in blood lipid levels, which are associated with the onset of various chronic diseases. Specifically, the levels of TGs, total cholesterol (TC), and low-density lipoprotein cholesterol (LDL-C) tend to increase, while high-density lipoprotein cholesterol (HDL-C) concentrations become relatively irregular during aging [43,44]. In humans and mice, plasma TG levels have been found to increase with age, thereby leading to its potential as a biomarker of aging due to its positive correlation with age [45,46,47,48]. By characterizing the metabolic changes in young and old mice, aging has been shown to be accompanied by an increase in the FFA levels [49]. In rats, plasma cholesterol levels also have an age-dependent increase that can be mitigated by regulating lipoprotein metabolism [50]. Regarding lipoprotein levels, both low-density lipoproteins (LDLs) and LDL-C have been reported to increase with age [51]. The determinant of plasma lipoprotein concentrations is affected by the rate at which lipoproteins are removed from the plasma. Additionally, the efficiency of lipoprotein plasma removal is reduced in the elderly [52]. High-density lipoproteins (HDLs) play a preventive role in atherosclerosis by exporting cholesterol and promoting cholesterol metabolism. However, changes happened in the composition of the HDLs during aging. HDLs isolated from elderly patients were reported to contain less cholesterol [53]. In addition, HDL function was impaired in older subjects, and its effectiveness in promoting cholesterol efflux and inhibiting LDL oxidation decreased [54]. Taken together, these data make a strong case for blood lipid and plasma lipoprotein levels being highly relevant to aging.

### 3.2. Age-Related Changes in Triglyceride Metabolism

TGs can be obtained by both exogenous pathways (dietary intake) and endogenous pathways (de novo synthesis). Exogenous TGs are absorbed in the intestine, formed as chylomicrons, and released into circulation. The liver plays a crucial role in TG metabolism, as it can take up and enzymatically hydrolyze chylomicrons, thereby converting them into residual components [55]. Endogenous TGs are mainly produced in the liver through glycolysis. Glycerol triphosphate reacts with esterified coenzyme A, thereby leading to the formation of TGs. Following their transportation by lipoproteins and utilization by extrahepatic tissues, excess TGs are stored in adipose cells. During starvation or sympathetic activation, adipose triglyceride lipase (ATGL) catalyzes the TGs in adipocytes to produce one FA and diacylglycerol (DAG). The DAG is then hydrolyzed by hormone-sensitive lipase (HSL) to generate another FA and monoacylglycerol (MAG). Finally, the MAG is hydrolyzed by monoglyceride lipase (MGL) to release glycerol and the last FA. The FAs produced during this process are used to provide energy through beta-oxidation (β-oxidation), which occurs in mitochondria and involves a series of enzymatic reactions that break down FAs into acyl-CoA and ketone bodies (acetoacetate, acetone, and β-hydroxybutyrate). These acyl-CoAs are further oxidized to produce TGs and ATP.

#### 3.2.1. Fatty Acid Uptake and Triglyceride Synthesis Increase in Liver with Age

Studies have suggested that the liver lipid content in aged mice increases due to the internalization of remnant chylomicrons, which requires the absorption of circulating FAs by hepatic cells. FA uptake in 20-month-old rats resulted in a two-fold increase compared to 2-month-old rats, and this change was detected as early as in middle-aged rats (10-month-old) [56]. The FA transporter CD36 (CD36) on the hepatic cell membrane plays a central role in controlling the FA flux into the liver. A study showed that the index of CD36 in the liver biopsies of healthy elderly subjects increased significantly [57]. This finding was confirmed in older mice, with the gene and protein expression of CD36 being significantly increased in the aged group [57]. Additionally, the pregnane X receptor (PXR) induces liver CD36 mRNA expression, and PXR gene expression is positively associated with age, which may lead to increased FA uptake and liver TG accumulation with age [58,59].

Liver FAs can also be synthesized through de novo lipogenesis (DNL), and the genes responsible for liver DNL are upregulated during aging. The regulation of liver adipogenesis is primarily controlled by sterol regulatory element-binding protein-1c (SREBP-1c), which can increase the expression of other genes involved in adipogenesis, such as ATP citrate lyase (Acly), fatty acid synthase (Fasn), and stearoyl-CoA desaturase-1 (SCD-1). To identify the molecular mechanisms underlying increased FA synthesis in aged mice, researchers analyzed the expression of the hepatic lipogenesis genes, including SREBP-1c, Acly, Fasn, and SCD-1, and found that their expressions were significantly increased [46]. The expression of SREBP-1c is tightly regulated by nuclear receptor farnesoid X receptor (FXR) [60]. Studies have shown that the gene and protein expressions of FXR in the livers of older mice were decreased. The levels of the downstream target genes of FXR, such as small heterodimer partner (SHP) and bile salt export pump (Bsep), were also decreased [46]. These findings suggest that FXR regulates SREBP-1c in opposing directions and ultimately leads to increased TG synthesis with age.

Dietary patterns can impact the absorption and de novo synthesis of FAs in elderly individuals. The consumption of a high-fat diet (HFD) may lead to alterations in fat metabolism, thus further contributing to FA uptake and TG synthesis. Studies indicate that the gene and protein expressions of the CD36 in mice fed with a HFD were significantly higher than those in mice fed a normal diet, thereby suggesting that CD36-mediated liver FA uptake was enhanced [57]. Additionally, the expressions of SREBP-1c and acetyl-CoA carboxylase (ACC) genes were significantly increased in the liver of aged mice fed with a HFD, thereby activating the DNL pathway [61]. Conversely, the calorie restriction (CR) diet reduced the levels of CD36 and ACC protein expressions in elderly subjects fed a normal diet, thus equalizing their levels with those of the younger group [62].

#### 3.2.2. Mitochondrial Dysfunction Leads to Impairment of the β-Oxidation

During aging, lipid synthesis increases and lipid catabolism decreases, thereby disrupting lipid homeostasis. Mitochondrial FA β-oxidation is the primary pathway for FA degradation, wherein it plays a crucial role in maintaining the energy balance [63]. Evidence has shown that liver β-oxidation becomes weaker in aged mice compared to young mice under HFD conditions, as confirmed by the decreased levels of the end-product β-hydroxybutyrate (BHBA) in the liver [61]. This decrease in FA oxidation was also observed in another study, which found that levels of BHBA were reduced in the plasma, thereby indicating a decline in the ketogenic capacity in aging mice [49]. Acylcarnitines transport FAs into the mitochondria and are essential for β-oxidation to occur. An increase in the plasma acylcarnitine concentration with age has been observed in healthy individuals, thus suggesting that mitochondrial function was impaired and β-oxidation declined [64].

Peroxisome proliferator-activated receptor α (PPARα) is a major regulator of the β-oxidation in all organs, and its dysfunction can contribute to age-related diseases. Studies have shown that the up-regulation of the advanced glycation end product receptor (RAGE) in the livers of aging mice leads to the down-regulation of PPARα and the decreased expression of downstream target genes regulated by PPARα, such as carnitine palmitoyltransferase 1a (CPT1a), carnitine palmitoyltransferase 1b (CPT1b), and acyl-CoA dehydrogenase medium chain (MCAD), which explains the weakened mitochondrial FA oxidation and the development of age-related fatty liver diseases [65]. Similarly, a notable down-regulation of the PPARα and its downstream target proteins, including CPT1a and acyl-CoA oxidase 1 (ACOX1), were observed in the kidneys of 24-month-old rats. This down-regulation was attributed to the regulatory effects of the miR-21 and ultimately contributed to the progression of renal fibrosis [66]. Furthermore, the down-regulation of the genes involved in FA transport and oxidation was observed in the heart during aging, thereby leading to cardiac dysfunction [66]. Therefore, decreased FA oxidation during aging contributes to the occurrence of a variety of age-related diseases.

Interestingly, increased FA oxidation has been observed in a long-lived mouse model, thus indicating the importance of this process for organism lifespan extension [67]. Building upon this discovery, investigations have explored the effects of various compounds, including resveratrol and astragaloside IV, which have the ability to enhance FA oxidation and positively influence lipid metabolism in aged mice [68,69]. These exciting findings not only shed light on the mechanism underlying healthy aging, but also hold promise for the development of novel interventions aimed at improving overall health and longevity.

### 3.3. Age-Related Changes in Cholesterol Metabolism

There are two sources of cholesterol in the body: exogenous cholesterol, which can be obtained from the diet via NPC1L1, and endogenous cholesterol, which is primarily synthesized in almost all tissues, with the liver being the most important organ [70]. The cholesterol biosynthetic pathway comprises nearly 30 enzymatic reactions that convert acetyl coenzyme A to cholesterol. However, cholesterol cannot be completely oxidized into carbon dioxide and water in the body. The primary route of the excretion of cholesterol is from the liver, where it is converted to BAs and excreted in the feces. The other route is through the ABCG5/G8 receptors, which remove cholesterol directly and efflux it into the gallbladder [71]. Cholesterol that enters the gut is metabolized by intestinal bacteria to form steroids, which are then excreted in the feces.

#### 3.3.1. Endogenous Cholesterol Synthesis Increases during Aging

During aging, dietary cholesterol intake and endogenous cholesterol synthesis increase, which may contribute to the development of fatty liver disease. Studies have shown that hepatic cholesterol levels become elevated in senescence-accelerated-prone mice [72]. In a study on 28-month-old mice, the expressions of cholesterol-synthesis-related genes, such as glucose transporter 2 (GLUT2), glucokinase (GK), sterol regulatory element-binding protein-2 (SREBP-2), 3-hydroxy-3-methylglutaryl coenzyme A reductase (HMGCR), and 3-hydroxy-3-methylglutaryl-CoA synthase (HMGCS) were increased, and further cellular experiments showed that the reactive oxygen species (ROS) played an important role in this process [73]. In addition, other studies have found that the ROS leads to the complete activation of the 3-hydroxy-3-methylglutaryl coenzyme A (HMG-CoA) reductase, which is a key rate-limiting enzyme in cholesterol synthesis, thereby leading to the increase in cholesterol synthesis with age [74]. Furthermore, studies on aging rats have shown that the ROS can cause the dephosphorylation of the HMG-CoA reductase through the mitogen-activated protein kinase (p38/MAPK) pathway, thereby leading to its activation [75]. Since excessive cholesterol accumulation has harmful effects on the body, reducing cholesterol synthesis through interventions such as the mechanistic target of rapamycin (mTOR) and sirtuins is of great significance [71].

#### 3.3.2. Bile Acid Enterohepatic Circulation and Lipoprotein Dynamics Changes with Age

Excess cholesterol, particularly LDL-C, is a significant risk factor for atherosclerotic CVD. To mitigate this risk, the body has developed multiple mechanisms to remove excess cholesterol from circulation, including both LDL receptor (LDLr)-dependent and LDLr-independent mechanisms. The LDLr-dependent mechanism involves the binding and internalization of LDL particles for lysosomal degradation, while the LDLr-independent mechanism involves the conversion of cholesterol into BA via cholesterol 7alpha-hydroxylase (CYP7A1), which is then followed by metabolism via the gut microbiota. BAs produced by the liver and gut are recycled and reused through enterohepatic circulation, which helps to conserve BA reserves and maintain cholesterol homeostasis.

As was previously mentioned, LDL-C levels increase with age, which is associated with a decrease in the LDL-C clearance. Normally, LDL-C and very low-density lipoprotein cholesterol (VLDL-C) are removed from circulation by hepatic LDLr to regulate cholesterol homeostasis [76]. However, studies have shown that hepatic LDLr expression decreases with age in humans, thus leading to a decrease in the LDL-C clearance from circulation [77]. This decrease has also been observed in elderly mice, as both the gene and protein expressions of the LDLrs decreased with age [78]. The reduction in LDLr expression may be mediated by the adenosine monophosphate-activated protein kinase (AMPK) and the downstream SREBP-2/proprotein convertase subtilisin kexin type 9 (PCSK9)/LDLr signaling pathway. AMPK levels increases with age, and they can regulate lipid metabolism by affecting the expression of the downstream target gene SREBP-2 [79]. PCSK9 is regulated by SREBP-2, which promotes the degradation of LDLrs on the surface of hepatic cells [80,81]. The gene expression of SREBP-2 and the blood levels of PCSK9 have been shown to increase with age [73,82,83]. Therefore, the activation of the AMPK/SREBP-2/PCSK9/LDLr signaling pathway may account for the age-related decrease in the LDLr and LDL-C clearance. Moreover, in LDLr knockout mice, the plasma LDL levels still increased with age, thereby indicating that other factors besides the reduction in LDLrs contribute to the imbalance in cholesterol metabolism throughout the body during aging [84].

Furthermore, BA synthesis decreases with age in humans. Previous studies on biopsied livers in the fasting state have shown reduced CYP7A1 expression in the elderly [85,86]. Additionally, aging is inversely correlated with Cyp7a1 mRNA levels [87]. The decrease in BA synthesis may reduce the utilization of cholesterol in the body, which could lead to an increase in the plasma cholesterol levels [88]. The age-related decreases in BA synthesis and their potential impact on cholesterol utilization are closely linked to the observed disturbances in the enterohepatic circulation of BAs during aging, which are evidenced by the reduced intestinal reabsorption and liver transport of the BAs. Aging was associated with the decreased mRNA expression of the intestinal BAs reabsorption transporter Asbt [27]. The mRNA expressions of the liver BA uptake transporters Na+/taurocholate cotransporting polypeptide (Ntcp) and organic anion transporting polypeptide 1b2 (Oatp1b2) were decreased during aging, whereas the expression of the BAs efflux transporter Bsep remained unchanged in aging mice [27,89]. Moreover, there were gender-dependent changes in the expression of these BA transporters during aging in mice, with Ntcp and Oatp1b2 mRNA expressions being reduced in aging female mice, but remaining constant in aging male mice [90]. These gender-specific differences in transporter expression may be regulated by changes in the estrogen levels in female mice after middle age.

## 4. Lipid-Related Chronic Diseases in the Elderly

**Figure 2:**
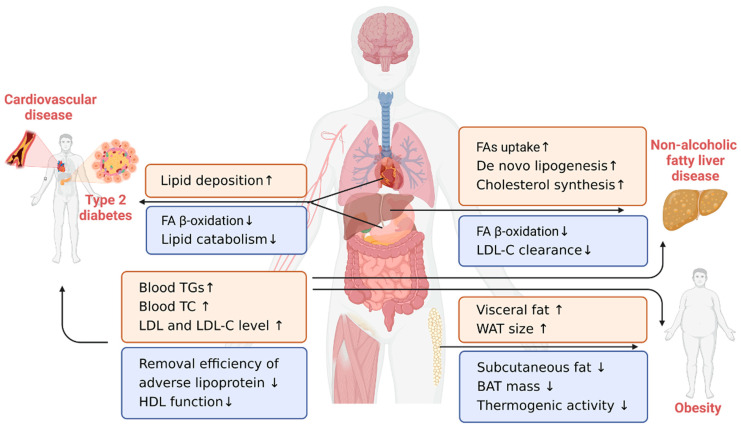
Key metabolic mechanisms of lipid dysregulation in the elderly and their associated chronic diseases. The body’s lipid homeostasis is maintained through a combination of hepatic anabolism and catabolism, adipose tissue storage, peripheral tissue utilization, and subsequent balance in blood circulation. These endogenous lipid metabolic changes in the elderly include increased lipid production and accumulation (corresponding to the red box) and decreased lipid consumption and clearance (corresponding to the blue box), which result in age-related chronic diseases such as cardiovascular disease, type 2 diabetes, nonalcoholic fatty liver disease, and obesity. ↑ means studies showing a significant increase; ↓ means studies showing a significant decrease; FA—fatty acid; TG—triglyceride; TC—total cholesterol; LDL—low-density lipoprotein; LDL-C—low-density lipoprotein cholesterol; HDL—high-density lipoprotein; WAT—white adipose tissue; BAT—brown adipose tissue. Figure 2 was created using Biorender with permission for publication from Biorender.com (accessed on 9 July 2023).

Lipid metabolism disorder is one of the key pathogenic factors for the occurrence and development of a series of lipid-related chronic diseases. In general, lipid-related diseases include CVD, T2D, NAFLD, and obesity, which seriously threaten public health [91]. Given that changes in lipid metabolism occur in healthy older adults, it is important to note that these changes may contribute to pathological alterations. Therefore, understanding the role of lipid metabolism in the development of these diseases may provide new insights into their underlying mechanisms and facilitate the development of effective treatments and prevention for the elderly (Figure 2).

### 4.1. Cardiovascular Disease

CVD is recognized as the primary cause of death in elderly individuals and is considered as a true disease of aging, with atherosclerosis being closely associated with lipid metabolism disorders [92,93]. Age-related disruptions in lipid metabolism result in the elevated levels of cholesterol, TGs, and LDLs in the bloodstream, thereby leading to lipid deposition beneath the inner lining of the blood vessels, which in turn forms atherosclerotic plaques. Evidence suggests that LDL-C, TC, and TG levels are positively correlated with the incidence of coronary heart disease, with the TC/HDL-C ratio being the strongest predictor of coronary heart disease in the elderly [2]. Thus, the continuous monitoring of these clinical markers is crucial during the aging process [92].

Furthermore, aging is associated with a greater tendency for lipid deposition in the heart and blood vessels. For instance, 25-month-old mice showed a significant accumulation of triacylglycerols in their heart tissues compared to 4-month-old mice [94]. Additionally, elderly mice exhibited higher rates of lipid deposition in their aortic arches than younger mice [95]. A study on mice has shown that the cluster of differentiation 73 (CD73), which is an exonuclease that catalyzes the conversion of adenosine monophosphate (AMP) to adenosine, can promote atherosclerosis on the vessel walls in aged mice by inhibiting lipid catabolism [96]. In addition, CD73 is an interesting molecule that could promote excess vascular lipid accumulation and accelerate atherosclerosis.

Preventing atherosclerosis requires a careful diet and exercise from a young age. High-TG and low-HDL-C levels at a young age are positively associated with accelerated epigenetic aging in midlife, thereby indicating that optimal lipid levels in early life may slow down epigenetic aging and delay the onset of diseases such as atherosclerosis [97]. Furthermore, older adults who engage in regular aerobic exercise have shown elevated HDL-C levels and decreased TC/HDL-C ratios, thereby suggesting that regular physical exercise can improve the lipid profile in older adults [43].

### 4.2. Type 2 Diabetes

T2D is a chronic metabolic disease that is prevalent among the elderly, wherein it is characterized by lipid metabolism disorders that lead to abnormal lipoprotein levels, which are often associated with diabetes progression. A study on Chinese older adults confirmed an independent inverse association between HDL-C levels and diabetes risk [98]. Additionally, the ratio of TG/HDL-C was positively correlated with T2D risk, and other lipid-related indicators have also been identified as risk factors for predicting T2D [99]. People with T2D have a significantly higher visceral adiposity index, which can serve as a predictor of T2D risk [100]. The triglyceride-to-glucose fasting index has also been considered as a supplementary indicator in diagnosing prediabetes in the elderly.

Studies have further shown that lipid metabolism disorders primarily affect glucose metabolism through insulin resistance, thereby leading to diabetes. Impaired lipid metabolism can contribute to the ectopic storage of adipocytes, thereby leading to insulin resistance occurrence or development [101]. Along with glucose intolerance, insulin resistance induces T2D during aging [102]. Research suggests that postprandial TG levels in T2D individuals are higher than in nondiabetic individuals due to insulin resistance in the intestinal epithelial cells. To improve the postprandial blood lipid and glucose indexes in T2D, researchers propose a focus on chylomicron metabolism [103].

### 4.3. Obesity

The increasing prevalence of obesity in the elderly population is a matter of concern [104]. Obesity can accelerate aging and contribute to the development of various age-related diseases, such as sarcopenia, T2D, CVD, and almost all lipid-related diseases, which pose a serious threat to the health of the elderly [105]. Research has indicated that the incidence of obesity in the elderly is associated with lipid metabolism disorders [106]. During the aging process, the activity of the sympathoadrenal system decreases, which may reduce lipid turnover rates, thereby leading to obesity in the elderly [107,108].

The distribution and function of adipose tissue change during the aging process. Older adults frequently experience an accumulation of visceral fat and a loss of subcutaneous fat, with an increase in white adipose tissue size and a decrease in brown adipose tissue function being the most noticeable changes [109]. Specifically, in older rats, adipocytes exhibit increased size, reduced DNL and lipolysis, and enhanced esterification [110]. Meanwhile, brown adipose tissue mass decreases, and its ability to produce heat weakens with aging, thereby leading to fat accumulation and the increased incidence of obesity in the elderly [111].

Adipose-derived stromal/stem cells (ASCs) play a critical role in energy balance maintenance, fat storage, and adipocyte homeostasis [112]. Studies have shown that the ability of ASCs to proliferate and differentiate decreases with age [113]. After transplanting the ASCs from young mice (2 months) into old mice (22 months), it was found that the plasticity of the stem cells, liver function, and lipid metabolism of the old mice improved, thereby indicating that ASCs had a positive effect on the lipid metabolism in aged animals and had the potential to be used as antiaging and antiobesity agents [114].

### 4.4. Nonalcoholic Fatty Liver Disease

NAFLD is a significant aging-related issue caused by the accumulation of excessive hepatocellular LDs, known as simple steatosis, which can progress to nonalcoholic steatohepatitis (NASH) and associated fibrosis [115]. Studies have indicated that NAFLD is prevalent in the elderly population in Asia, wherein it affects approximately 40% of this population and has the potential to develop into liver fibrosis with aging [116]. Similar observations have been reported in aging mice, wherein TG and cholesterol contents increased in the livers of aged mice [46,65,73].

As mentioned previously, studies have demonstrated that aging processes are likely to promote NAFLD through increased lipid synthesis and decreased lipid catabolism in the liver [46,65,73]. In addition, dietary fat intake significantly impacts the liver fat in aged mice. Under the condition of a HFD, the contents of neutral fat in the livers of aged mice increased compared with young mice. The contents of polyunsaturated FAs with liver-protective effects decreased, while the contents of saturated and monounsaturated FAs with lipotoxicity increased [61]. Thus, dietary restriction is protective against hepatocyte senescence and liver fat deposition in aged mice [117].

Furthermore, understanding the underlying mechanism and identifying effective therapeutic targets for age-related NAFLD is of great importance. Several potential targets have been identified in the context of age-related liver conditions. In the livers of aged mice, the levels of CDGSH iron–sulfur domain-containing protein 2 (Cisd2) decreased by approximately 50%, thus resulting in increased de novo hepatic fat synthesis and accumulation. Cisd2 is also a promising target for slowing down liver aging [118]. Additionally, previous experiments showed that, during the aging process, hepatic lipid deposition reduced in zinc finger gene 1 (JAZF1)-transgene mice, which was possibly due to the decreased gene expressions involved in adipose storage, including SREBP-1c, SCD-1, and fatty acid synthase (FAS) [119]. This suggests that JAZF1 may be another potential target, which could prevent lipid production.

## 5. Conclusions

Fundamentally, lipid metabolism is maintained through a delicate balance between lipid intestinal digestion and absorption, systemic synthesis, and catabolism. This review summarizes the adverse changes in lipid metabolism that occur during aging and their relationship with the development of age-related chronic diseases. The insights from this review have promising implications for developing nutrition and health guidelines regarding the distinct lipid metabolism needs of the aging population. The process of conducting a literature review has revealed inconsistencies in the age-related research findings, which are possibly due to increased individual differences in the aging process. Genetic variations, lifestyle choices, environmental exposures, and health conditions all influence aging outcomes. Addressing this issue requires rigorous, larger-scale prospective or interventional studies. In addition, utilizing more advanced approaches, such as single-cell sequencing, spatial transcriptomics, and proteomics could offer profound insights into the underlying mechanisms of age-related lipid metabolism disorders and may reduce research discrepancies.

Recent progress has shown that the aging process affects not only one aspect of lipid metabolism, but every regulatory component of it. Furthermore, lipid-related diseases are likely to be interconnected, with the occurrence of one disease increasing the risk of others. Further research is needed to explore the connections between these regulators and their interactions in causing age-related diseases. Accumulating evidence has shown that a nutritionally adequate diet is beneficial for all age-related diseases, and dietary intervention may be a new target for preventing excess fat deposition and for treating lipid-related diseases in the elderly. Overall, this review offers valuable insights into the prevention of nutrition-related chronic diseases in the elderly, which is beneficial for promoting a healthy lifespan.

## Data Availability

Not applicable.
